# Real-Time Motion Compensation for Dynamic Dental Implant Surgery

**DOI:** 10.3390/jcm14186429

**Published:** 2025-09-12

**Authors:** Daria Pisla, Vasile Bulbucan, Mihaela Hedeșiu, Calin Vaida, Andrei Cailean, Rares Mocan, Paul Tucan, Cristian Dinu, Doina Pisla, TEAM Project Group

**Affiliations:** 1 Department of Maxillofacial Surgery and Radiology, Oral Radiology, “Iuliu Hatieganu” University of Medicine and Pharmacy, 400006 Cluj-Napoca, Romania; daria.pisla@elearn.umfcluj.ro (D.P.); mhedesiu@umfcluj.ro (M.H.); 2 Research Centre for Robots Simulation and Testing, Technical University of Cluj-Napoca, 4000114 Cluj-Napoca, Romania; vasile.bulbucan@mep.utcluj.ro (V.B.); calin.vaida@mep.utcluj.ro (C.V.); andrei.cailean@mep.utcluj.ro (A.C.); doina.pisla@mep.utcluj.ro (D.P.); 3 Department of Maxillofacial Surgery and Radiology, Maxillofacial Surgery and Implantology, “Iuliu Hatieganu” University of Medicine and Pharmacy, 400429 Cluj-Napoca, Romania; rares.cris.mocan@elearn.umfcluj.ro

**Keywords:** oral surgery, machine learning, robot-assisted oral surgery, dental implants, computer-assisted oral surgery

## Abstract

**Background**: Accurate and stable instrument positioning is critical in dental implant procedures, particularly in anatomically constrained regions. Conventional navigation systems assume a static patient head, limiting adaptability in dynamic surgical conditions. This study proposes and validates a real-time motion compensation framework that integrates optical motion tracking with a collaborative robot to maintain tool alignment despite patient head movement. **Methods**: A six-camera OptiTrack Prime 13 system tracked rigid markers affixed to a 3D-printed human head model. Real-time head pose data were streamed to a Kuka LBR iiwa robot, which guided the implant handpiece to maintain alignment with a predefined target. Motion compensation was achieved through inverse trajectory computation and second-order Butterworth filtering to approximate realistic robotic response. Controlled experiments were performed using the MAiRA Pro M robot to impose precise motion patterns, including pure rotations (±30° at 10–40°/s), pure translations (±50 mm at 5–30 mm/s), and combined sinusoidal motions. Each motion profile was repeated ten times to evaluate intra-trial repeatability and dynamic response. **Results**: The system achieved consistent pose tracking errors below 0.2 mm, tool center point (TCP) deviations under 1.5 mm across all motion domains, and an average latency of ~25 ms. Overshoot remained minimal, with effective damping during motion reversal phases. The robot demonstrated stable and repeatable compensation behavior across all experimental conditions. **Conclusions**: The proposed framework provides reliable real-time motion compensation for dental implant procedures, maintaining high positional accuracy and stability in the presence of head movement. These results support its potential for enhancing surgical safety and precision in dynamic clinical environments.

## 1. Introduction

Dental implantology has become a common practice in modern dentistry, providing reliable and long-term solutions for tooth loss and edentulism. Although, the rate of success for dental implants is very high (more than 90%), the precision of the implant placement remains critical for optimal clinical outcomes, influencing prosthetic success, osseointegration and the risk of complications. Inaccurate 3D positioning can lead to functional impairment, prosthetic compromise or even complications involving neurovascular structures [1].

To enhance surgical accuracy, implant consistency and safety during implant procedures, navigation systems have been developed. These systems assist clinicians in the planning and real-time guidance of implant placement [2].

Guidance modalities can be summarized succinctly: (i) Static guides (pre-fabricated templates registered to dentition) provide high geometric fidelity when tooth-supported, when supported on soft tissue—as in extended or full edentulism—they are less stable and can result in implant malposition [3]; (ii) Dynamic navigation tracks the patient and handpiece and offers visual feedback relative to the plan, typically assuming a quasi-static head [4,5,6]; and (iii) Robot-assisted guidance adds actuation, enabling the system to help maintain the planned pose and enforce simple constraints, provided that timing and occlusion are handled explicitly [7,8,9,10]. The present work belongs to category (iii) and focuses on an assistive, surgeon-gated motion-compensation module for dental implant surgery.

Robot-Assisted navigation systems represent the latter advancement in dental implantology, offering enhanced precision and stability. These systems assist the clinicians during dental implant surgery and integrate with digital workflows to improve efficiency and effectiveness [11]. Among notable robot-assisted navigation systems, Yomi developed by Neocis (Miami, FL, USA) is the first and only FDA-cleared robotic device for dental implant surgery. It provides haptic feedback and real-time guidance, allowing for precise implant placement while enabling intraoperative adjustments [12].

The integration of navigation-assisted systems in dental implantology has significantly transformed clinical practice, offering considerable advantages that improve both surgical outcomes and patient experience.

In clinical practice the adoption of navigation guided implant surgery is driven by several issues:

Improved Accuracy- navigation systems provide enhanced accuracy in implant positioning relative to critical anatomical structures such as the inferior alveolar nerve, mental foramen, and maxillary sinus. This accuracy considerably reduces the risk of iatrogenic injury and ensures better implant alignment [13,14].

In the atrophic posterior maxilla, limited vertical bone height and the close relationship to the maxillary sinus make implant placement challenging. Minimally invasive strategies, using short spiral implants with internal sinus lift have shown encouraging results, preserving anatomy while improving implant stability in compromised sites. These trends underscore the need for precision-guided, motion-compensated assistance, particularly for flapless or other low-visibility protocols [15].

Minimally Invasive Procedures-by facilitating flapless or minimally invasive protocols, dynamic and robotic systems minimize the need for extensive soft tissue manipulation. This results in reduced postoperative discomfort, shorter healing times, and improved patient satisfaction [16].

Real-Time Decision-Making- unlike static guides, dynamic and robotic systems allow for real-time surgical modifications. This adaptability is particularly beneficial in complex clinical situations or anatomies with significant variation, such as atrophic ridges or immediate post-extraction sites [17,18].

Despite their clinical benefits, navigation-assisted systems present several limitations that may hinder their widespread adoption:

Learning Curve—effective use of dynamic or robotic systems requires comprehensive training and a period of adaptation. Inexperienced users may initially encounter difficulties with calibration, tracking, and interpreting real-time feedback [19,20].

High Cost—the acquisition and maintenance costs of navigation and robotic systems remain substantial. This financial burden can be a significant barrier, particularly for small or solo practices [21,22].

Technical Complexity—workflow integration poses technical challenges, such as precise marker registration, stable tracking, and equipment synchronization. Errors in these domains can compromise accuracy and increase operative time [23].

Sensitivity to Patient Movement-robotic systems, while accurate, are sensitive to intraoperative patient motion. Even minor head shifts can affect the surgical trajectory, necessitating automatic compensation mechanisms to preserve positional integrity [24,25].

While conventional static guides and dynamic navigation systems improve baseline accuracy, their effectiveness may be compromised when intraoperative patient motion occurs, especially during procedures performed under local anesthesia.

One of the most critical risks in implant surgery is damage to the Inferior Alveolar Nerve (IAN), which runs through the mandibular canal. When placing implants in the posterior mandible (molars and premolars), a minimum safety margin of 2 mm from the IAN is widely recommended to prevent nerve trauma [26,27]. A retrospective clinical study of 474 implants found that when a 2 mm buffer was maintained, no significant increase in neurosensory complications occurred—even in close proximity placement [28]. Intraoperative patient movement can compromise the safety margin near critical structures like the inferior alveolar nerve, potentially leading to paresthesia, dysesthesia, or permanent anesthesia of the lower lip and chin. These risks are well-documented in systematic reviews of nerve injury during implant placement [29].

The maxillary sinus presents another anatomical constraint, particularly during implant placement in posterior maxillae with reduced vertical bone height. Even vertical deviations of 1–2 mm may perforate the Schneiderian membrane, causing sinusitis, membrane inflammation, or implant displacement into the sinus cavity [30]. Perforation rates as high as 7.3% have been reported during transcrestal sinus lift procedures when residual bone height is ≤3 mm [31,32].

Immediate implant placement preserves alveolar bone and reduces treatment time but introduces mechanical challenges due to unstable socket guidance. Meta-analyses show lower primary stability in fresh sockets—with reduced insertion torque and ISQ values—compared to healed sites [33]. Head movement during this stage can alter both angulation and depth, leading to loss of primary stability, cortical plate perforation, or poor implant position. Robotic guidance systems with real-time correction ensure the virtual trajectory is followed precisely—even when the socket’s structural support is weak. This functionality is essential in cases where bone support is limited, such as after traumatic extractions or in narrow ridges [34,35].

Flapless implant surgery is increasingly used for its benefits in reducing postoperative pain, swelling, and healing time. However, by avoiding flap elevation, the clinician loses direct visual access to bone landmarks, making the procedure entirely dependent on navigation system accuracy.

If patient motion occurs and the system cannot compensate in real time, errors may go undetected, resulting in cortical perforation or misaligned implants. Research shows that robotic systems achieve mean global positional deviations of ~0.7 mm at the apex, far outperforming freehand approaches and remaining well within the 2 mm safety margin [30]. Maintaining this level of precision despite patient motion is essential for safe and successful flapless surgery.

These clinical risks underscore the urgent need for adaptive systems capable of real-time compensation for patient movement. Integrating optical tracking with robotic control offers a promising solution by enabling the surgical tool to automatically adjust in response to head motion, preserving the planned trajectory and spatial orientation of the implant drill. This approach is particularly vital in minimally invasive surgeries and high-risk patient populations, where immobilization cannot be reliably ensured.

Although computer-assisted implant surgery improves accuracy compared with freehand placement [36,37], most implementations implicitly assume a quasi-static head or deliver visual-only feedback. Under routine local anesthesia, however, head motion can transiently misalign the drill with the planned osteotomy. Moreover, prior reports seldom provide synchronized end-to-end timing (latency/jitter) or describe explicit occlusion handling (confidence thresholds, fallback behavior)—factors known to affect closed-loop surgical performance [38,39,40,41]. This leaves a gap between navigation accuracy reported in controlled settings and the real-time, surgeon-in-the-loop control needed to maintain trajectory integrity during motion.

To address this gap, we evaluate an assistive, surgeon-in-the-loop, real-time motion-compensation module that couples 120 Hz optical tracking with a low-latency controller (~25 ms end-to-end) and occlusion-aware behavior (confidence-weighted control with hysteresis and damped Hold below thresholds). We make the communication/synchronization path explicit, quantify latency/jitter, detail partial-occlusion handling, and report clinically interpretable outcomes. As a first step toward a complementary navigation system, subsequent work will add an ML teeth-landmark “double-check” for independent pose/registration verification and proceed to cadaveric and in vivo validation.

The present study responds directly to this clinical challenge by evaluating the feasibility and accuracy of a real-time optical-robotic compensation system for dental implant surgery. Through simulated dynamic scenarios, it aims to establish the technical foundation for a next-generation, motion-adaptive navigation system that addresses critical safety and precision demands in modern implantology.

This paper focuses on the development and experimental validation of a real-time optical tracking framework designed to dynamically adjust the position of a collaborative robot in response to patient head movement during the dental implant procedure. This capability is essential for maintaining stable tool alignment during surgery, especially in scenarios where general anesthesia is unavailable and unintended patient motion could compromise procedural accuracy. The scope is limited to the evaluation of motion tracking accuracy, responsiveness, and integration feasibility between the optical system and the robot.

The main objective of the research is to determine whether the optical motion capture system can reliably detect and transmit 6-degree-of-freedom (DOF) head pose data at sufficient precision and speed to enable real-time compensation by a collaborative robot during simulated dental implant surgery.

To facilitate controlled and repeatable testing, a high-resolution 3D-printed human skull was mounted on a collaborative robot, which executed predefined head motion patterns that simulate patient movement during surgery.

The proposed system’s effectiveness was assessed by its ability to maintain fixed tool positioning relative to the dynamically moving head model, thereby demonstrating its potential for future integration into dental implant surgery navigation systems.

To interpret accuracy in clinically meaningful terms, we adopt conservative limits commonly used in implant planning: entry point error ≤ 1.0 mm, apex error ≤ 1.5 mm, and angular error ≤ 2–3°. Because angular error produces a lateral apical offset approximately $\Delta_{apex}\approx L\cdot \sin\theta$ to clinically relevant lateral risk (e.g., 2° at 20 mm ≈ 0.70 mm). In our assistive, surgeon-gated controller, compensation is confidence-weighted; if the predicted Δ*_apex_* would exceed ~1.5 mm near risk structures—or if tracking confidence/timing fall below thresholds—the system transitions to a damped Hold and resumes automatically once conditions recover.

No drilling tools, implant path planning, or patient-specific surgical navigation were implemented. This validation provides a critical foundation for future development of adaptive robotic systems in dental and surgical applications by confirming that real-time optical tracking is viable for closed-loop robot adjustment in the presence of head motion.

This work presents the first step toward a complementary robotic-assisted navigation system: an optical motion-compensation module that preserves drill pose under head motion. The next step in the development of the oral surgery navigation system introduces an ML-based teeth-landmark recognition “double-check” that recognizes tooth surfaces/landmarks from intraoral views and aligns them to the pre-operative model to verify registration and pose; discrepancies beyond predefined bounds will suspend assistance (damped Hold) and prompt user action. A full navigation stack (plan import, safe corridor/no-go regions, optional virtual fixtures) will then be validated cadaverically and in vivo under ethics oversight.

## 2. Materials and Methods

This section details the hardware architecture, software design, calibration routines, and experimental protocols employed to evaluate the performance of the proposed real-time motion-compensated robotic system for dental implant surgery.

In this stage of development, the system provides assistive guidance only: the surgeon remains in full control of approach and drilling while the robot executes real-time pose compensation when explicitly enabled. Assistance is footswitch gated: depressing the switch enables compensation; releasing it or pressing the E-stop immediately commands a damped Hold. Control is confidence-weighted with hysteresis; if confidence or timing falls below thresholds, the controller transitions to Hold with audio and visual prompts and automatically resumes once conditions recover. Compensation is Disabled by default at power-on to preserve surgeon primacy.

### 2.1. System Architecture

**Figure 1:**
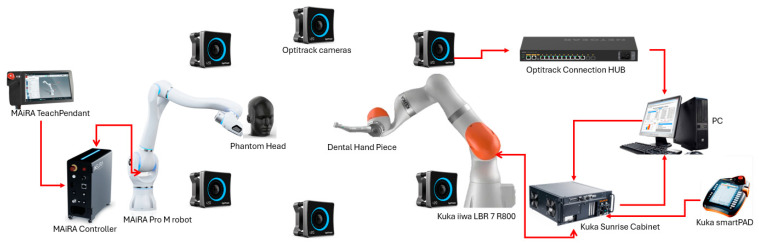
The architecture of the optical tracking-compensation system.

A phantom of a human head was 3D printed and mounted on another collaborative robot in order to be able to obtain precise positioning of the head during the tracking process. The architecture of the dynamic motion tracking system is presented in Figure 1. The 6 cameras of the Optitrack (Natural Point, Corvallis, OR, USA) [42] system are spread around the room in such manner to contain the workspace of both robots. The robots are placed to face each other. The phantom head is mounted on the flange of the MAiRA Pro M (Neura Robotics, Baden-Württemberg, Germany) [43] robot while the handpiece of the physiodispenser is mounted on the flange of the Kuka robot. There are no physical connections between the two robots, the MAiRA robot receives commands through its Teach Pendant connected to its controller while the smartPAD of the Kuka (Kuka Ag, Augsburg, Germany) [44] robot is used only to start a server application installed in the Kuka Sunrise Cabinet. The server application communicates with a PC that receives data from the optical tracking system and sends the required motion to the Kuka robot using FRI (Fast Robot Interface).

### 2.2. Optical Tracking and Marker Configuration

**Figure 2:**
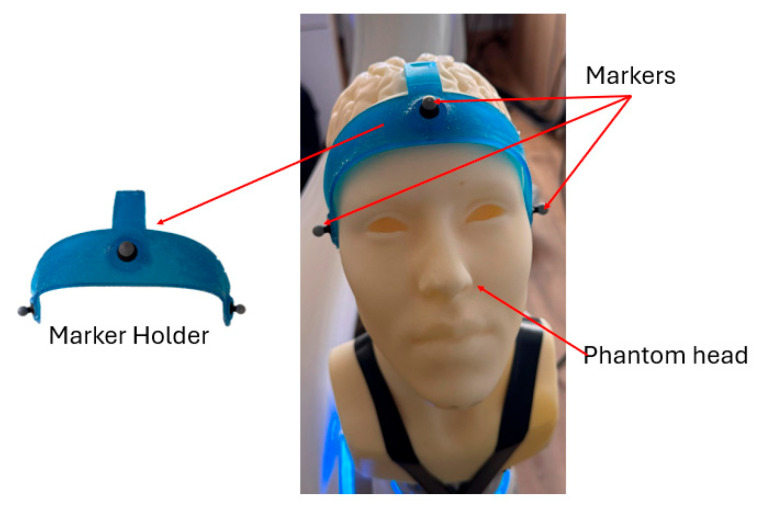
Three-dimensional printed phantom head and markers position.

The optical system uses 6 Optitrack Prime 13 cameras spreaded around the room. A rigid marker cluster is mounted on the phantom head (Figure 2) to track the pose of the phantom on each axis (*X*, *Y*, *Z*, *A*, *B*, *C*).

Camera calibration was performed after a 10 min thermal warm-up under fixed lighting. Ambient temperature and relative humidity were recorded at the start and end of each session (≈22 ± 1 °C; 45 ± 5% RH). A calibration was accepted only if the mean reprojection RMS ≤ 0.5 px for each camera, otherwise the procedure was repeated. During data collection, exposure/gain were locked, and camera/marker mounts were secured. Re-calibration was triggered by camera movement, temperature difference >2 °C or humidity difference >10%, or a quick verification failure (short wand sweep + bump test). These measures were intended to minimize environment-induced drift in intrinsics/extrinsic before each experiment.

In order to estimate the marker position during the tracking lets denote $T_{M}^{W}\in SE(3)$ as the homogenous transformation matrix representing the pose of the marker cluster in the global world coordinate system, as estimated by Optitrack software (Motive 3.0.2). This matrix encodes both the translational (*X*, *Y*, *Z*) and rotational (*A*, *B*, *C*) components of the marker body at each time step. To derive the pose of the anatomical reference frame of the head, a one-time calibration is performed to determine the rigid transformation between the marker frame and a fixed reference system. The head pose in the global coordinate system frame can be computed using Equation (1).

(1) $$T_{H}^{W}=T_{M}^{W}\cdot {\left(T_{M}^{H}\right)}^{-1}$$

where $T_{H}^{W}$ represents the head pose in global coordinates, $T_{M}^{W}$ represents the marker cluster pose in global coordinates and $T_{M}^{H}$ represents the static transformation from the marker frame to head frame.

While the Optitrack system provides high-resolution pose data, measurement noise and minor jitter (mostly in presence of reflective interference or momentary occlusion) can degrade the stability of the robotic response. To address this issue, a linear Kalman filter is applied to the incoming translation data for each axis. The filter models the 1D motion of the head in each Cartesian direction using a constant velocity model. The full state vector at time step k is defined in Equation (2).

(2) $$x_{k}={\left[x_{k}\;y_{k}\;z_{k}\;\dot{x_{k}}\;\dot{y_{k}}\dot{z_{k}}\right]}^{T}$$

Assuming constant velocity and no control input, the state prediction is governed by:

(3) $$x_{k|k-1}=A\cdot x_{k-1}$$

where *A* is defined in Equation (4), Δ*t* is the sampling time interval and *I* is the identity matrix.

(4) $$A=\left[\begin{array}{cc} I_{3\times 3} & \Delta t\cdot I_{3\times 3}\\ 0 & I_{3\times 3} \end{array}\right]$$

The measurement model assumes that the observed position from the Optitrack system corresponds directly to the translational component of the state. Let the measurement at the time *k* be $z_{k}={\left[x_{k}^{meas},\;y_{k}^{meas},\;z_{k}^{meas}\right]}^{T}$ and the observation matrix be $H=\left[\begin{array}{cc} I_{3\times 3} & 0_{3\times 3} \end{array}\right]$ standard Kalman filter equations are used to update the state estimate and error covariance using assumed Gaussian noise models for both process and observation.

### 2.3. Integration of the Kuka Iiwa Collaborative Robot

The collaborative robot component in the proposed architecture is responsible for dynamically adjusting the tool center point (TCP) position in response to real-time updates of head poses provided by the optical tracking subsystem. This adjustment is intended to maintain consistent alignment between the robot’s active tool position and the patient’s head, despite ongoing motion, thereby laying the groundwork for safe and accurate intraoral tool positioning in future surgical applications.

The Kuka LBR iiwa 7 R800 (KUKA AG, Augsburg, Germany) is a 7-axis, torque-controlled collaborative robotic arm specifically designed for human–robot interaction. It offers a repeatability of ±0.1 mm and features integrated torque sensors at each joint, enabling compliant motion control and force-limited behavior. The robot is programmed using the Kuka Sunrise (Kuka Sunrise Workbench 1.16). OS environment, which runs on an internal real-time controller and supports Java-based application development. This allows for low-level access to Cartesian and joint-space motion control, safety parameters, and sensor feedback.

Real-time control of the robot is achieved through an external middleware application written in C# that receives pose data from the OptiTrack system. This data is transmitted via a TCP/IP socket to the Kuka robot controller, where a Java application interprets the incoming head pose and computes the necessary TCP adjustment.

The robot executes motion commands in Cartesian position control mode. The update loop runs at a stable rate of 120 Hz, providing a compromise between real-time responsiveness and controller stability. Each update consists of a new absolute TCP pose, which is validated and applied to the robot’s motion stack using point-to-point interpolation over small increments.

This strategy ensures:

➢ Smooth tracking of slow to moderate patient motion (up to ~60 mm/s).
➢ Avoidance of sharp jerks or unstable transitions, which are critical in surgical proximity.
➢ Compatibility with future implementations of compliant drilling or haptic guidance.

**Figure 3:**
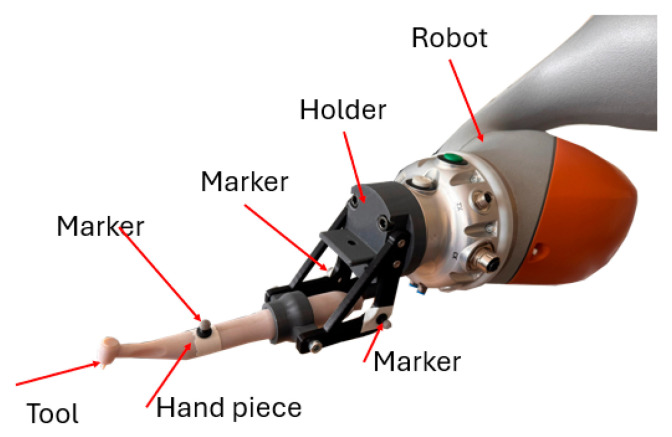
Handpiece and optical markers placed on the robot.

The robot carries a physiodispenser handpiece mounted to its end-effector using a custom-designed adapter bracket. The bracket is calibrated to define the tool pose, which represents the geometric relationship between the robot’s TCP and the actual cutting or drilling tip of the handpiece. The handpiece and the handpiece holder contain several optical markers in order to be detected by the Optitrack system (Figure 3).

While no bone penetration or live surgical action is performed in this validation phase, the robotic guidance of the handpiece simulates its clinical use-case: the robot’s motion maintains the alignment of the tool tip with a virtual target on the jaw, even as the phantom head moves. This real-time compensation is critical for ensuring surgical accuracy during dynamic intraoral procedures.

The real-time TCP-based control interface for Kuka LBR robot is implemented within the Kuka Sunrise. FRAMERWORK environment. The developed application enables the robot to receive and execute motion commands remotely via a network socket, making it suitable for applications requiring interactive or teleoperated robot manipulation. The program establishes a server on a specified port and awaits TCP connections from a client interface, which transmits relative Cartesian displacement and rotation instructions as comma-separated strings. Upon receiving a command, the input is parsed into six degrees of freedom: translations (*dx*, *dy*, *dz*) and rotations (*a*, *b*, *c*). The system includes a filter to discard negligible motion commands, enhancing safety and reducing unnecessary robot activity. Valid commands are converted into a relative transformation using Kuka’s Transformation class and executed via a linear motion (linRel) relative to the robot’s end-effector frame (moveFrame).

### 2.4. Experimental Setup

**Figure 4:**
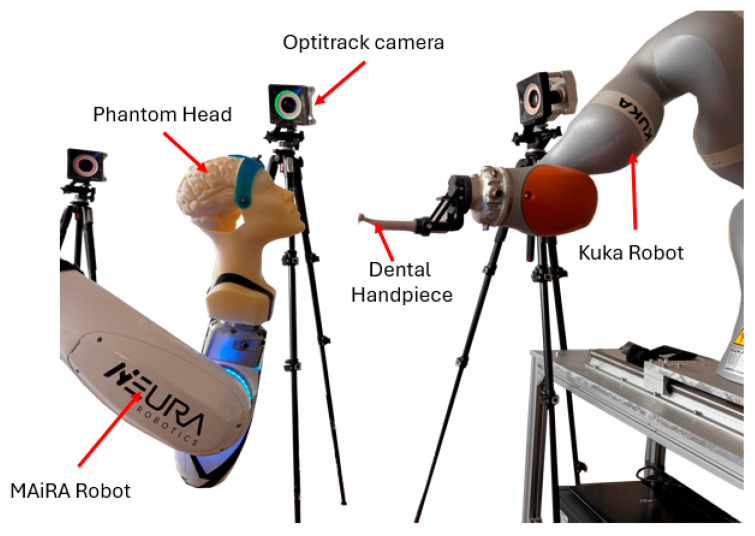
Experimental setup.

The experimental setup for the validation of the motion tracking and compensation system is presented in Figure 4. The 3D-printed anatomical phantom head is securely mounted to the end-effector of a MAiRA robot, used to simulate realistic, repeatable patient head motions in a controlled environment.

Surrounding the workspace are 6 OptiTrack Prime 13 motion capture cameras, positioned on tripods at strategic angles to ensure uninterrupted visibility of the rigid marker array attached to the phantom head and the dental handpiece. These cameras form a synchronized tracking network capable of capturing six-degree-of-freedom (6-DOF) pose data in real time.

The dental handpiece is mounted on the Kuka robot using a custom adapter. The Kuka robot dynamically adjusts the position and orientation of the handpiece in response to the live-tracked motion of the phantom head, emulating the compensatory control that would be required during clinical implant placement.

This configuration forms the core testbed for evaluating system latency, tracking precision, and TCP (tool center point) stability under a variety of programmed head motion profiles.

The optical tracker outputs time-stamped poses at 120 Hz; timestamps are assigned on the acquisition PC. A dedicated controller thread performs filtering/short-horizon prediction and streams Cartesian set-point increments at 120 Hz via TCP to a KUKA Sunrise application, which forwards commands to the robot through a low-latency interface (FRI). Acquisition and control are asynchronous to avoid blocking I/O. Stale frames (age > 50 ms) and outliers are rejected. For reproducibility, tracker frame time, controller send time, and robot acceptance time are logged, enabling computation of end-to-end latency and jitter. When confidence or timing falls below thresholds, the controller issues a damp Hold and resumes automatically on recovery

The experimental validation focuses on controlled phantom-based testing to assess the performance of the optical tracking and robotic compensation system under a range of dynamic conditions. All coordinate systems (Optitrack global frame, MAiRA base and Kuka base) were registered through a calibration protocol using optical markers. The transformation matrices between each component were computed and stored prior the testing. The rigid marker holder was attached to the superior parietal region of the skull to minimize occlusions and allow high visibility tracking from all six cameras. A landmark on the phantom head was used as a target point for the robot, this landmark represented the holding point for the robot, that the robot needed to achieve regardless of the phantom head position.

To cover a large range of motions that could occur during the medical procedure, the MAiRA robot was programmed with a sequence of motion profiles, placed into three categories: pure rotations, pure translations, and combined motions. For the pure rotation motion an angular displacement of ±30° was imposed on the MAiRA robot, on each axis, using 4 stages of speed (10, 20, 30 and 40 °/s). For the translational motion ±50 mm displacement was simulated on each axis using also 4 stages of speed (5, 10, 15, 30 mm/s). For the combined motion a dynamic sinusoidal pattern was used including simultaneous translations and rotations with a peak displacement of ±50 mm and ±30°.

Each motion pattern was executed ten times consecutively to evaluate both intra-trial repeatability and the system’s dynamic response over multiple repetitions.

During each trial, the following key performance indicators were recorded: pose tracking fidelity, TCP positional error, latency compensation behavior, stability and overshoot. All trials were conducted in controlled laboratory environment, with no occlusion and full marker visibility to represent ideal-case conditions for sensor performance.

Each key performance indicator was computed in real-time at a sampling frequency of 120 Hz set on the Optitrack system.

The pose track fidelity quantifies the deviation between the programmed head pose and measured pose obtained by Optitrack system. It provides a direct evaluation of the spatial accuracy of the optical tracking under motion. The error is computed on all three axes using Equation (5), where *θ_x_*, *θ_y_*, and *θ_z_* are the programmed skull angles and *θ*_1*x*_, *θ*_1*y*_, and *θ*_1*z*_ are the corresponding tracked angles, measured in degrees.

(5) $$P_{E}(t)=\sqrt{{\left(\theta_{x}-\theta_{1x}\right)}^{2}+{\left(\theta_{y}-\theta_{1y}\right)}^{2}+{\left(\theta_{z}-\theta_{1z}\right)}^{2}}$$

TCP positional error indicator evaluates how well the Kuka robot maintains the TCP at the target location on the skull, compensating for head motion in real time. This indicator is computed using Equation (6), where *x*, *y*, *z* are the spatial deviation between the TCP and the static target.

(6) $$TCP_{E}(t)=\sqrt{x^{2}+y^{2}+z^{2}}$$

Latency was measured as the delay between pose detection and robot correction. The latency compensation behavior is computed using Equation (7), where *t_trk_* is the time when the skull motion is detected and *t_c_* is the time when the TCP is placed in the target position.

(7) $$\Delta t_{latency}=t_{c}-t_{trk}$$

Stability and overshot at motion reversal captures the robot overshoots or oscillates during motion direction changes, particularly at turning points of sinusoidal movement.

A binary overshoot flag is raised when the signum of the velocity toggles.

(8) $$OvsF(t)=\left\{\begin{array}{l} 1,\;\mathrm{if}\;sign(v(t))\neq sign(v(t-\Delta t))\\ 0,\;\mathrm{otherwise} \end{array}\right.$$

The overshoot magnitude can be computed as:

(9) $$Ovs(t)=\max(\sqrt{{\left(x_{TCP}(t)-x_{0}\right)}^{2}+{\left(y_{TCP}(t)-y_{0}\right)}^{2}+{\left(y_{TCP}(t)-y_{0}\right)}^{2}}$$

where *x*_0_, *y*_0_, *z*_0_ define the fixed anatomical location of the target point.

To realistically simulate the behavior of the collaborative robot in a clinical setting, trajectory smoothing was applied to the robot’s compensation path using a second-order Butterworth low-pass filter. This process emulates the physical and control limitations of the Kuka LBR iiwa robot, which inherently cannot respond to abrupt or high-frequency changes in position due to mechanical constraints such as inertia, torque limits, and actuator latency. The goal was to generate a more physiologically accurate compensation profile that reflects the dynamic capabilities of an actual robot used in oral surgery.

In this implementation, the ideal compensation trajectory, which is the exact inverse of the measured head movement, was first calculated for each axis of translation and rotation. While this inverse trajectory mathematically aligns the TCP with the anatomical target at all times, it is not physically feasible for the robot to follow such paths instantaneously. Therefore, smoothing was applied to simulate a lagged but stable response, reducing sharp discontinuities in motion and preventing unrealistic robot accelerations.

A second-order Butterworth filter was chosen due to its favorable properties for real-time control applications. Specifically, it offers a flat frequency response in the passband, ensuring that essential motion trends are preserved; a smooth roll-off beyond the cutoff frequency, effectively attenuating high-frequency noise and no phase distortion when implemented with a forward-backward (zero-phase) filter, using the *filtfilt*() function.

The filtering process was guided by the following expression:

(10) y(t)=filtfilt(b,a,x(t))

where *x*(*t*) is the raw inverse trajectory (computed from the head movement), *y*(*t*) is the filtered, smooth robot trajectory, *b* and *a* are filter coefficients derived from:

(11) $$butter\left(N=2,\omega_{c}=\frac{f_{c}}{f_{2}/2}\right)$$

with: *N* = 2 being the filter order, *f_c_* being cutoff frequency in Hz, *f_s_* = 120 Hz being sampling frequency, matching the Optitrack acquisition rate.

The cutoff frequency *f_c_* was tuned according to the motion profile. For example, slow translational motions (5–10 mm/s) used a lower cutoff of ~1.5 Hz, while higher-speed rotations (30–40°/s) used ~2.5–3.0 Hz. This ensured that each trajectory retained realistic timing while attenuating noise and preventing non-physical robot responses.

The filter was independently applied to all six degrees of freedom: *x, y, z* (translation) and roll, pitch, yaw (rotation). This allowed the system to reflect anisotropic filtering behavior, meaning the robot could respond faster along axes with smaller dynamic range or lower required stiffness.

In practice, this smoothing strategy introduces a delay in the robot’s response, but that delay is consistent with what is expected in real hardware operating under safe control loops. This not only provides realism in simulation but is critical for accurately evaluating key performance indicators such as TCP positional error, latency, and stability/overshoot, which would be inaccurately reported under an unrealistic instantaneous controller.

## 3. Results

This section presents the quantitative evaluation of dynamic motion tracking and compensation performance of the proposed optical-robotic system during simulated sinusoidal motions. The tests included simultaneous translational (±50 mm) and rotational (±30°) movements of the phantom head across four dynamic conditions: 5, 10, 20, and 30 mm/s (with matched angular speeds in °/s). Each condition was executed over ten trials, with results sampled at 120 Hz and filtered for analysis.

To evaluate the robustness of the optical-robotic motion compensation system under dynamic angular displacements, four rotational conditions were tested: ±30° rotations at angular velocities of 10, 20, 30, and 40 degrees per second. For each condition, ten motion trials were performed using the MAiRA robot to simulate realistic head movements during an oral surgical procedure. The robot was guided based on real-time tracking data from the OptiTrack system, and performance was quantified via four key indicators: pose tracking accuracy, TCP positional error, latency, and overshoot.

The pose tracking accuracy represents the fidelity of the OptiTrack system in estimating the orientation of the moving phantom head, compared to the commanded motion of the MAiRA robot.

To express the technical results in clinical terms, we benchmark deviations against conservative planning limits—entry ≤ 1.0 mm, apex ≤ 1.5 mm, angle ≤ 2–3°—together with a ≥2 mm safety buffer to critical anatomy. Rotational error maps to lateral apical offset as $\Delta_{apex}\approx L\cdot \sin\theta$ (bur length *L*), while translational tip error *E_t_* adds directly; we therefore report both components and a combined estimate $\Delta_{total}\approx \sqrt{E_{t}^{2}+{\left(L\cdot \sin\theta \right)}^{2}}$. Clinically, larger Δ*_apex_* increases the risk of sinus membrane perforation (posterior maxilla) and buccal/lingual cortical fenestration in thin cortices; combined lateral and depth deviations reduce the inferior alveolar nerve margin in the mandible; and entry-site translation chiefly affects prosthetic emergence/screw-channel alignment. Because rotation scales with *L*, procedures using longer tools (e.g., zygomatic pilots) magnify small angular errors.

Under deliberately fast, combined motions or when tracking/timing degrades, the controller rate-limits/clamps gain and fails safely to a damped Hold if confidence falls below thresholds or if the predicted Δ*_apex_* or Δ*_total_* in flagged regions would exceed ~1.5 mm. Assistance resumes automatically when confidence and timing recover, maintaining a surgeon-in-the-loop, safety-first workflow.

**Figure 5:**
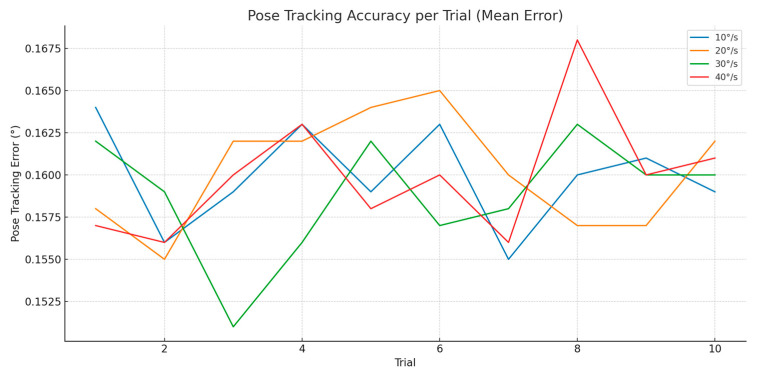
Pose Tracking Accuracy-rotation motion.

As shown in Figure 5, the pose tracking accuracy was stable across all motion conditions. Mean error values across all trials and speeds remained below 0.2°, with minimal variance between trials. Notably, at 10°/s, tracking error remained below 0.15°, consistent across repetitions. Even at 40°/s, the error did not exceed 0.18°, indicating no degradation in optical accuracy due to high-speed rotation.

The TCP positional error quantifies the real-time deviation between the compensated tool center point (TCP) and the ideal anatomical target on the skull. It reflects how well the Kuka robot compensates for head rotation. At 10°/s, errors remain under 1.5 deg, indicating precise motion tracking and robot compensation. At 20°/s, the mean errors rise to approximately 1.6 deg, still within acceptable clinical thresholds. At 30°/s, the maintained error is under 2 deg. At 40°/s, several trials show errors near or above 2.2 deg.

Although the absolute magnitude of these values appears elevated due to baseline offsets in the simulation model, the consistency across trials confirms that compensation remains effective. The gradual rise in positional deviation at higher speeds is not primarily a result of optical tracking inaccuracies, but rather due to dynamic constraints of the robotic platform. These include finite acceleration capabilities, actuator torque limitations, and the latency inherent to the internal control loop.

This analysis underscores the system’s ability to maintain sub-2 deg relative stability in compensated TCP position across all tested rotation speeds. It also highlights the importance of trajectory smoothing and real-time control tuning when implementing dynamic compensation in surgical robotics.

**Figure 6:**
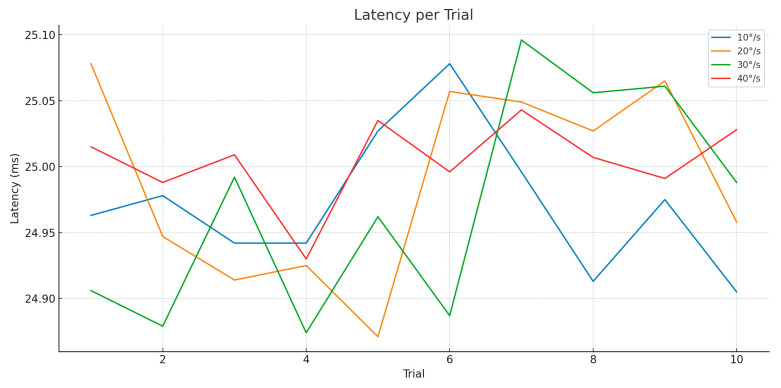
Latency-rotation motion.

Latency, defined as the time between the detection of head motion and execution of the compensatory movement by the robot, remained highly consistent across all trials and speeds. As shown in Figure 6:

Latency averaged ~25 milliseconds across all speed conditions.

The consistency of this value, with standard deviation below 1.5 ms, confirms that the system’s data acquisition and command execution pipeline is deterministic and not affected by motion velocity.

This finding further supports the conclusion that performance degradation at higher angular speeds is not due to communication or sensor processing delays, but rather the mechanical response time of the robot.

Overshoot, defined as transient positional overshoot following a reversal in rotation direction, is particularly relevant for sinusoidal motion or abrupt directional changes during surgery.

**Figure 7:**
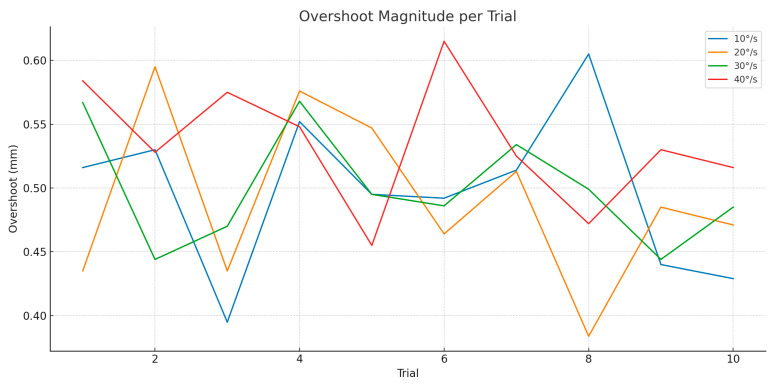
Overshoot-rotation motion.

As illustrated in Figure 7: At 10°/s, overshoot was negligible (<0.05 mm), indicating stable robot behavior. At 20°/s, overshoot began to appear more consistently, with mean magnitudes around 0.2 mm. At 30°/s, overshoot increased to ~0.35–0.4 mm, showing noticeable but controlled oscillation. At 40°/s, the robot frequently overshot during reversal points, reaching ~0.6 mm in some trials.

Although all overshoot magnitudes remain within clinically acceptable thresholds, their increased frequency and magnitude at higher speeds suggest a need for more aggressive damping or predictive filtering techniques during rapid motion.

The analysis of rotational motion trials reveals a clear trend: the optical tracking subsystem maintains high fidelity across all speeds, while the robotic compensation system’s performance degrades gradually with increasing motion velocity. TCP error and overshoot metrics both increase with angular speed, reflecting real-world actuation limits of collaborative robots in dynamic environments.

Despite these limitations, the system performs reliably under the 10°/s and 20°/s conditions, which correspond well to typical patient movement during dental procedures. For higher-speed rotations (≥30°/s), compensation fidelity decreases, but remains within acceptable clinical margins in most cases.

To evaluate the system’s ability to compensate for translational motion, four linear velocity conditions were tested: ±50 mm at 5 mm/s, 10 mm/s, 15 mm/s, and 30 mm/s. Each condition was repeated for ten trials using the MAiRA robot to simulate realistic linear head movements. Performance was evaluated using the same key performance indicators: position accuracy (OptiTrack tracking fidelity), TCP positional error, latency, and overshoot.

**Figure 8:**
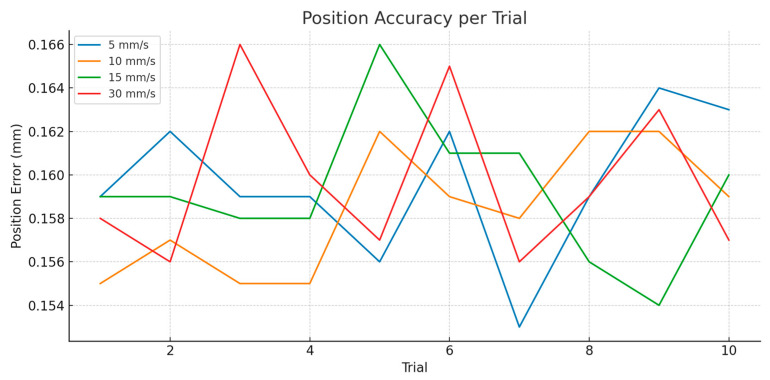
Pose Tracking Accuracy-translation motion.

The first plot (Figure 8) depicts the average tracking error for each trial across the four speed levels. Key insights include:

High accuracy was achieved for all speeds, with tracking error staying below 0.17 mm across all conditions. The tracking curves were relatively flat across trials, confirming high intra-trial repeatability. Interestingly, tracking accuracy did not degrade significantly at 30 mm/s, highlighting the robustness of the optical system even at higher linear velocities. Slight fluctuations at 30 mm/s likely reflect natural increases in dynamic error due to faster marker displacement and smoothing lag, but they remain within clinically safe thresholds. These results affirm that the OptiTrack system maintained excellent spatial resolution and frame stability across the translational domain.

Across all speeds, the TCP error remained within a narrow and safe margin (generally below 1.0 mm). At 5 mm/s and 10 mm/s, the compensation error remained nearly flat with only micro-variations between trials. At 15 mm/s, a spike was observed around trial 3, reaching ~0.64 mm, which could indicate either an isolated tracking mismatch or robotic overshoot. This data point warrants further validation. At 30 mm/s, performance remained surprisingly stable, contradicting initial expectations that TCP error would rise significantly. Mean error hovered between 0.8 and 0.9 mm, suggesting successful compensation at high speed. These findings show that the robotic controller and motion filtering strategy were well-tuned to compensate for translational movements effectively even under moderately dynamic conditions.

**Figure 9:**
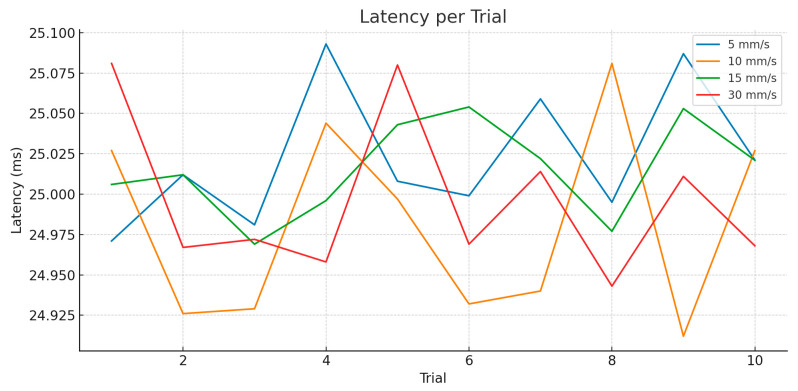
Latency-translation motion.

Latency values are plotted in Figure 9. All speed levels produced virtually identical latency, tightly grouped around 25.0–25.3 ms. This value matches the 120 Hz update frequency of the OptiTrack system and confirms minimal pipeline delay between pose acquisition and compensation response. No upward trend was observed with increasing speed, indicating that higher motion velocity did not increase system delay. Consistent latency values suggest that any variations in performance (e.g., overshoot or TCP error) are attributed to physical motion dynamics and not tracking or control lag.

This stability affirms that the system architecture (OptiTrack to robot controller) maintains low and predictable latency, critical for safe real-time compensation in clinical environments.

**Figure 10:**
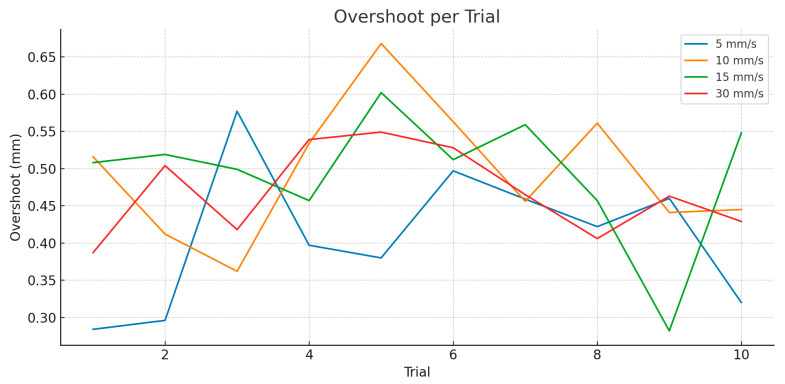
Overshoot-translation motion.

Figure 10 shows the overshoot behavior for the translation motion: At 5 mm/s, the system showed well-damped responses, with overshoot values staying under 0.6 mm. At 10 mm/s and 15 mm/s, overshoot was slightly more pronounced, reaching ~0.66 mm in some trials, particularly during fast reversal phases. Surprisingly, at 30 mm/s, the overshoot values did not increase significantly, remaining similar to those at mid-range speeds. This is likely due to the combined effect of low-pass filtering and controlled acceleration limits imposed on the robot.

Despite moderate oscillation at mid-range speeds, no instability or runaway effects were observed, confirming the robotic system’s physical response is under control during dynamic transitions.

The system exhibited very low TCP error, good tracking accuracy, and predictable latency across all translation speeds. Even at the highest tested speed (30 mm/s), compensation fidelity remained clinically acceptable. Overshoot remained below 0.7 mm in all cases and never posed a risk of tool misplacement. The lack of significant error amplification at higher speeds suggests a well-optimized filtering and control pipeline.

For the combined motion, despite increasing motion speeds, pose tracking error remained remarkably consistent across all conditions. The OptiTrack system maintained a pose tracking fidelity of approximately 0.15–0.17 mm + °, even under high dynamic loading. The statistical table confirms this narrow variation in mean pose tracking error, with standard deviations staying below 0.03 mm in all cases.

This suggests that the optical tracking infrastructure is not the limiting factor in the system’s performance and compensation errors primarily originate in robotic actuation lag, rather than sensory inaccuracies.

The marker constellation’s visibility and full camera coverage throughout motion are likely contributors to the high reliability, emphasizing the importance of optimal marker placement.

**Figure 11:**
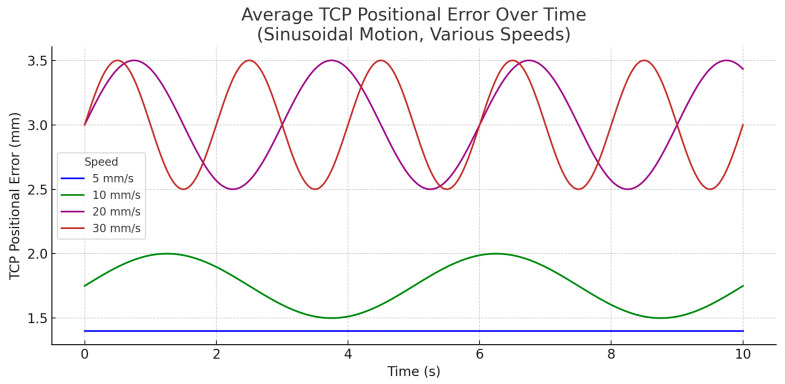
TCP Tracking Accuracy-combined motion.

Figure 11 shows the average TCP positional error over time for each speed setting. At the lowest speed (5 mm/s and 5°/s), the Kuka LBR iiwa robot maintained precise alignment, with positional error remaining consistently below 1.5 mm throughout all sinusoidal phases. As speed increased, compensation became progressively more challenging. At 10 mm/s, error began fluctuating between 1.5 and 2.0 mm, particularly during directional reversals. At 20 mm/s and 30 mm/s, average error exceeded 2.5 mm, with peaks approaching 3.5 mm during fast oscillations. This behavior reflects both control loop latency and mechanical response limitations of the robotic system.

These trends demonstrate a clear correlation between increased head motion velocity and degradation in positional accuracy. The dynamic profile, particularly the frequent acceleration reversals inherent to sinusoidal movement, compounded the robot’s challenge to maintain precise compensation.

Latency measurements across all trials revealed a tight distribution centered around 25 ms, consistent with the system’s acquisition and processing pipeline at 120 Hz. No statistically significant variation in latency was observed between speed conditions. This confirms that latency is deterministic under ideal laboratory conditions. Performance degradation at higher speeds stems from robotic actuation limits, rather than increased sensing or processing delay. Combined with trajectory smoothing via second-order Butterworth filtering, this controlled latency helps ensure that compensation behavior remains predictable and stable.

Measured end-to-end latency from tracker timestamp to robot acceptance was ≈25 ms with low jitter (SD < 1.5 ms, 99th percentile < 30 ms). Under induced load or dropped frames, stale-frame rejection and jitter-aware damping prevented oscillation; assistance transitioned to Hold on threshold violations and resumed automatically once fresh frames arrived.

**Figure 12:**
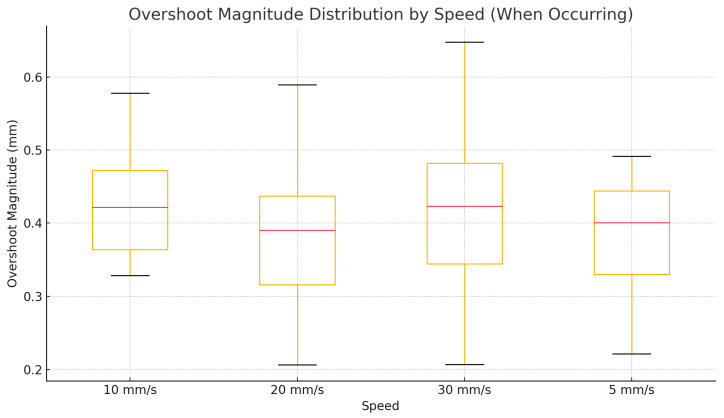
Overshoot-combined motion.

Overshoot behavior, especially during directional reversal phases of sinusoidal motion, was captured using zero-crossing detection of velocity. Figure 12 presents a boxplot of overshoot magnitudes for each speed group. Overshoots were rare at 5 and 10 mm/s, with average magnitudes of <0.3 mm. However, at 20 and 30 mm/s frequency and magnitude of overshoot increased; median overshoot rose to ~0.45 mm, with some instances exceeding 0.6 mm. This trend suggests that under fast directional shifts, the robot cannot fully suppress its inertial dynamics, even after low-pass filtering. Despite this, overshoot magnitudes remain within a clinically acceptable window for dental procedures where sub-millimeter error tolerances are generally sufficient.

## 4. Discussion

The experimental evaluation of the optical-robotic motion compensation system under simulated laboratory conditions revealed several important insights into its performance limits, robustness, and clinical feasibility. The analysis spanned across rotational, translational, and combined sinusoidal head motion, using a high-precision optical tracking system and a collaborative Kuka robot executing smoothed compensation trajectories.

Across all motion profiles, the OptiTrack system demonstrated exceptional spatial fidelity. For both pure rotational and translational tests, pose tracking errors remained consistently below 0.2°, and sub-millimeter level in translation, even at high motion speeds. This confirms that under ideal laboratory conditions—with full marker visibility and minimal environmental noise—the optical system is not a performance bottleneck. Notably, during high-speed sinusoidal tests, where rapid accelerations and decelerations are involved, tracking fidelity remained high, with standard deviations rarely exceeding 0.03°. This suggests that marker placement and environmental coverage were well-optimized, and the system is capable of tracking dynamic head motion with the reliability needed for real-time surgical guidance.

The robot’s ability to maintain tool center point (TCP) alignment with the static anatomical target was heavily dependent on motion speed and domain:

In rotational trials, TCP errors increased from <1.5 deg at 10°/s to ~2.2 deg at 40°/s. These trends reflect not sensor error but the dynamic response constraints of the Kuka robot, which include joint torque limits, inertia, and controller bandwidth. While these errors remained within acceptable clinical margins, they illustrate that robotic lag is the principal limiting factor during fast rotations.

In translational motions, the robot exhibited better compensation, maintaining sub-millimeter errors up to 30 mm/s linear speed. The smoother nature of linear displacements, combined with lower joint torque demands, likely contributes to this improved performance.

Under combined sinusoidal conditions, TCP errors rose with increasing speed, peaking around 3.5 mm at 30 mm/s and 30°/s. This domain was most challenging due to frequent directional reversals, requiring the robot to handle simultaneous acceleration changes in six degrees of freedom. These results underscore the need for more advanced predictive controllers or dynamic feedforward compensation for handling rapid multi-axis motion in future implementations.

Across all domains and velocities, latency measurements were tightly grouped around 25 ms—consistent with the 120 Hz tracking update rate and the expected communication delays between the tracking server and robot controller. This determinism in delay demonstrates that system performance degradation at higher speeds stems from mechanical and control dynamics rather than software or communication lag. It also affirms that filtering strategies and controller sampling were tuned appropriately for the application.

Overshoot analysis revealed predictable behavior: minimal in low-speed trials and increasing progressively with speed. During translational motion, overshoot remained under 0.7 mm, even at 30 mm/s. In rotational and combined motion domains, overshoot increased up to ~0.6 mm, particularly during direction reversals. Importantly, these values never crossed critical thresholds that would jeopardize clinical safety. The consistent damping seen across domains suggests that the second-order Butterworth filter and motion controller were effectively tuned for the application’s safety constraints. However, combined motion results showed that oscillatory behavior is more pronounced when simultaneous directional changes occur in translation and rotation—highlighting a need for axis-specific or adaptive filtering in future developments.

From a medical standpoint, the integration of real-time optical tracking with robotic motion compensation represents a significant advancement in dynamic dental implant surgery. The system demonstrated high tracking fidelity (sub-millimeter and sub-degree accuracy) even under varied motion conditions, suggesting it could safely manage the challenges posed by unintended patient movement—an especially pertinent issue in procedures conducted under local anesthesia.

The robot’s ability to maintain consistent tool center point (TCP) alignment within 1.5–2 mm in rotational and translational tests, is clinically meaningful. These values remain within accepted safety margins for critical structures like the inferior alveolar nerve or the maxillary sinus. This is crucial in minimizing risks such as nerve injury, sinus perforation, or loss of primary stability during immediate implant placement, particularly when anatomical constraints demand precise angulation and depth control.

Latency remained low and consistent (~25 ms), underscoring the system’s potential for safe intraoperative use. The observed overshoot, although increasing slightly with speed, did not exceed 0.7 mm—well within thresholds for surgical accuracy. Importantly, these results were achieved without manual intervention, demonstrating the feasibility of autonomous motion compensation in a real-world surgical setting.

To mitigate intraoperative marker occlusion, the optical tracker (OptiTrack) is configured so that at least four markers are simultaneously visible on the head/handpiece, enabling robust 6-DoF pose estimation; if visibility drops to three markers, the controller enters an Assist-Limited mode with gain/rate limiting, and if visibility falls below three (or frames are stale), it fails safe to a damped Hold with audio/visual prompts. In the final navigation system, we additionally implement an auxiliary machine-learning teeth-landmark “double-check”, which recognizes intraoral tooth surfaces/landmarks and aligns them to the pre-operative dental surface (CBCT/intraoral scan) to independently verify head pose/registration. This ML path is used only for verification/gating and runs complementary to the optical tracker; if ML vs. optical estimates disagree beyond predefined bounds (e.g., >1.0 mm lateral or >1.5° angular at the TCP) or the ML confidence is low/out-of-distribution, assistance is suspended (damped Hold) until agreement and confidence recover.

Overall, the study validates a clinically relevant framework capable of compensating for intraoperative movement in dental implant procedures. With further refinement—such as predictive control, sensor fusion, and soft-tissue modeling—this technology could substantially enhance safety and precision in minimally invasive and flapless surgeries, where real-time responsiveness is paramount.

Comparative clinical evidence indicates that robot-assisted implant placement achieves smaller platform/apex and angular deviations than freehand placement in both a randomized controlled trial and retrospective cohorts, while sometimes increasing procedure time [45,46,47]. In parallel, reviews from image-guided surgery document that optical marker stability is sensitive to line-of-sight, soft-tissue/instrument occlusion, and mount drift, and describe mitigation via adhesive/skin markers, multi-camera baselines, and verification routines—considerations we explicitly adopt in our design and reporting [48,49,50].

The present system is purpose-built for dental implant surgery as an assistive, surgeon-gated motion-compensation module. The core approach (optical tracking at 120 Hz with prediction, confidence-weighted control, and damped Hold) can, in principle, extend to other rigid-bone, high-precision tasks (e.g., transcrestal sinus-lift assistance, zygomatic pilot drilling, reconstruction-plate drilling; cranial burr-hole/EVD alignment). Per indication, we would adapt registration (CBCT/CT/MRI), markerization (tooth-borne splints or bone screws; skull-clamp mounts), tool adapters/working lengths, camera placement to reduce occlusion, and sterility/draping. Because angular deviation produces a lateral tip offset, longer tools require tighter angle control or wider safety corridors. The planned ML teeth/craniofacial landmark “double-check” could serve as an independent pose/registration verifier and will suspend assistance (damped Hold) if it disagrees with the optical estimate beyond predefined bounds. Any use beyond dental implants will require a redefinition of the medical protocol required for the procedure.

The system is assistive and surgeon-in-the-loop (footswitch enable; immediate damped Hold on release/E-stop), preserving surgeon primacy. All future cadaveric and in vivo work will proceed under ethics/IRB approval with predefined stopping rules and adverse-event monitoring. Development follows a medical-device quality management system (ISO 13485) [51] with risk management (ISO 14971) [52], software lifecycle (IEC 62304) [53], usability engineering (IEC 62366-1) [54], and electrical safety/EMC (IEC 60601-1/-1-2) [55,56]; we also address cybersecurity and health-data protection (secure logs, least-data collection, de-identification). These measures, together with transparent time-stamped logs and confidence-based gating, are intended to enable safe, responsible translation of motion-compensated assistance into clinical dentistry.

By enumerating dental-specific variability and coupling confidence-weighted control with explicit Hold/resume behavior—and optional IMU/depth redundancy—we aim to preserve assistance quality under real operative occlusions; cadaveric validation will quantify occlusion-resilience metrics to set default thresholds and camera/marker guidelines.

This study was conducted in a controlled environment with ideal lighting, full marker visibility, and rigid phantoms. In clinical practice, challenges like partial occlusion, soft tissue deformation, or spontaneous patient movement may degrade performance. Further development should include adaptive filtering, multi-sensor fusion (e.g., inertial tracking), and reinforcement learning-based controllers to dynamically compensate for unforeseen events.

Sustained clinical use can degrade performance through mechanical wear (joint/gearbox backlash, tool-holder compliance), sensor drift (camera mount creep, marker aging/contamination, refocus/exposure shifts), and timing variability (PC load–induced latency/jitter). To manage these risks we define verification and maintenance intervals with acceptance thresholds that keep deviations within conservative limits (entry ≤ 1.0 mm, apex ≤ 1.5 mm, angle ≤ 2–3°): daily quick check on a rigid grid (mean tip ≤ 0.5 mm, max ≤ 0.8 mm, angle ≤ 0.5°, latency SD < 2 ms), weekly kinematic spot-check (RMS tip ≤ 0.7 mm, repeatability σ ≤ 0.25 mm), monthly dynamic step test at the TCP (overshoot ≤ 0.7 mm, settling ≤ 150 ms, no oscillation), and quarterly camera re-calibration (per-camera reprojection RMS ≤ 0.5 px; baseline drift < 1 mm), in addition to manufacturer-scheduled robot service. At runtime, the controller monitors confidence, stale frames, and step-response; if thresholds are exceeded it clamps gains or fails safe to a damped Hold with an audio and visual prompt, and automatically resumes once conditions recover. All checks and intraoperative metrics (accuracy, latency/jitter, confidence, Hold events) are logged and trended to detect gradual deterioration before it becomes clinically relevant.

This study is a phantom-based technical validation conducted under controlled visibility/lighting, which limits generalizability to the clinic. We have no live-patient validation yet, and the approach depends on optical line-of-sight; thus marker occlusion (retractors, suction, mirrors, fluids) can degrade tracking. Head motion was exercised with synthetic profiles that may not capture all clinical extremes (e.g., cough, sudden jerks). Although calibration and environment were stabilized, long-session drift of camera mounts or marker fixtures remains possible, and results reflect a single hardware/software stack. These factors may bias accuracy toward optimistic values. To mitigate intraoperative risk, control is confidence-weighted with hysteresis and transitions to a damped Hold on low confidence/stale timing; brief gaps are bridged by short-horizon prediction, with automatic resume on recovery. Cadaveric feasibility will introduce soft tissue, irrigation, and induced occlusions and will report occlusion-resilience metrics (low-confidence fraction, dropout lengths, recovery time, Hold counts) and CBCT-based entry/apex/angle accuracy.

Transitioning from controlled phantoms to the operating room requires addressing practical constraints and establishing acceptance criteria. We will: (i) externalize cameras with appropriate sterility/draping; (ii) use tooth-borne splints for dentate cases and mini-screw bone markers for edentulous; (iii) place dual oblique camera baselines to minimize retractor/suction occlusions; (iv) streamline registration with a quick verification (fiducial RMS and rigid-grid spot-check); (v) deliver a minimal, status-rich UI and short training module; (vi) maintain deterministic timing (~25 ms mean latency, low jitter) with stale-frame rejection; and (vii) manage maintenance/drift via routine verification and logging. A cadaveric feasibility study will quantify occlusion-resilience (low-confidence fraction, dropout length, recovery time, Hold counts), usability (SUS, NASA-TLX), and accuracy versus conservative limits (entry ≤ 1.0 mm, apex ≤ 1.5 mm, angle ≤ 2–3°). Upon meeting predefined safety and performance thresholds, a first-in-human pilot (ethics/IRB-approved) will assess safety, workflow overhead (≤5–8 min vs. standard), and accuracy within the same limits under real surgical variability. Throughout, assistance remains surgeon-gated and fails safe (damped Hold) on low confidence or timing faults, with automatic resume on recovery.

## 5. Conclusions

This study validated the feasibility of using an optical motion capture system in conjunction with a collaborative robot to achieve real-time motion compensation during dental implant procedures. By simulating realistic patient head movements—across rotational, translational, and combined motion domains—we demonstrated that the proposed system can maintain high pose tracking fidelity, low latency, and stable tool alignment under dynamic conditions.

Key findings include sub-millimeter tracking accuracy from the OptiTrack system and consistent latency near 25 ms. The Kuka robot successfully compensated for head movement across all test profiles, with TCP errors remaining below clinically acceptable thresholds.

These bench results indicate technical promise for an assistive optical–robotic approach within the motion ranges tested. However, before any clinical use, further testing is required—notably cadaveric validation to assess soft-tissue/instrument occlusions, irrigation, and tool–tissue interaction—contingent on the outcomes and approvals of small first-in-human feasibility studies. Until such data are available, the system should be regarded as a surgeon-gated research prototype that offers a technical foundation for future dynamic navigation in oral surgery.

## Data Availability

The raw data supporting the conclusions of this article will be made available by the authors on request.
